# *Foxp3* Expression in Liver Correlates with the Degree but Not the Cause of Inflammation

**DOI:** 10.1155/2011/827565

**Published:** 2011-05-25

**Authors:** Matthaios Speletas, Nikoletta Argentou, Georgios Germanidis, Themistoclis Vasiliadis, Konstantinos Mantzoukis, Kalliopi Patsiaoura, Pavlos Nikolaidis, Vaios Karanikas, Konstantinos Ritis, Anastasios E. Germenis

**Affiliations:** ^1^Department of Immunology and Histocompatibility, Medical School, University of Thessaly, Biopolis 41110 Larissa, Greece; ^2^First Department of Internal Medicine, AHEPA Hospital, Aristotle University of Thessaloniki, 54636 Thessaloniki, Greece; ^3^Gastroenterology and Hepatology Division, Hippokration Hospital, Aristotle University of Thessaloniki, 54642 Thessaloniki, Greece; ^4^Department of Pathology, Hippokration Hospital, 54635 Thessaloniki, Greece; ^5^First Department of Internal Medicine, Medical School, Democritus University of Thrace, 68100 Alexandroupolis, Greece

## Abstract

Patients with chronic viral hepatitis display increased expression of *Foxp3* in liver, suggesting that Tregs expansion contributes to persistent infection. The purpose of this study was to elucidate whether the expression of *Foxp3* relates not to the viral infection but to the resulting liver inflammation. Liver biopsies obtained from 69 individuals (26 chronic HBV hepatitis, 14 chronic HCV hepatitis, 11 nonalcoholic fatty liver disease, 8 autoimmune diseases, 2 methotrexate-related toxicity, and 8 controls) were examined, by qRT-PCR, for the mRNA expression of *Foxp3*, *IL-10*, *TGF-*β*1*, *Fas, FasL, TRAIL, caspase-3, TNF-*α*, IFN-*γ*,* and *IL-1*β**. Significant increase of *Foxp3* was observed in all disease groups compared to controls, which was positively correlated with the intensity of inflammation. The expression of the apoptosis mediators *Fas, FasL*, and *TRAIL*, but not of *IL-10* and *TGF-*β*1*, was also significantly elevated. Our findings indicate that, independently of the initial inducer, liver inflammation is correlated with elevated expression of apoptosis mediators and is followed by local Treg accumulation. Further research towards the elucidation of the underlying casual relationships is required, in order to clarify whether our results signify the existence of a uniform Treg-mediated regulatory mechanism of apoptosis-induced inflammation.

## 1. Introduction

T regulatory cells (Tregs) are important mediators of immune suppression, and their presence prevents reactions against self by inducing regulatory signals to antigen presenting cells (APCs) and/or effector T cells [1, 2]. Their ablation increases the risk of autoimmunity [3] whilst, on the contrary, their signals could also affect nonautoreactive clones, leading to inhibition of antineoplastic, antimicrobial, antiparasitic, and antiviral immune responses [1, 4, 5]. Accumulating evidence indicates that patients with chronic viral hepatitis display increased numbers of Tregs (both natural and inducible) in peripheral blood [6–8] or liver [9–11], which in turn exert a suppressive function against specific hepatitis C virus- (HCV-) or hepatitis B virus- (HBV-) T effector clones in vitro [6–10]. Thus, it has been suggested that the expansion of Tregs during viral hepatitis may contribute to an inadequate immune response, causing persistent viral infection. However, the precise role of Tregs in the pathogenesis of chronic hepatitis is the subject of intense debate, since it has been demonstrated that Tregs suppress the function and the expansion of virus-specific T effector cells ex vivo, irrespective of the patients having chronic or resolved virus infection [11, 12].

Herein, we describe an mRNA expression study in biopsy material scheduled to further elucidate the role of Tregs in the pathogenesis of liver damage in chronic viral HBV and HCV hepatitis. Bearing in mind that recent structural and molecular studies suggest apoptosis, rather than necrosis, being the mechanism of liver cell death in chronic viral hepatitis [13, 14], the expressions of forkhead box P3 (*Foxp3*) gene, characterizing naturally occurring CD4^+^ Tregs (nTregs) [1], as well as that of inteleukin-10 (*IL-10*) and transforming growth factor-*β*1 (*TGF-β1*), characterizing Tr1 and Th3 inducible Tregs (iTregs) [15], respectively, were examined in relation to the expression of major apoptosis mediators, namely, *Fas,* Fas Ligand (*FasL*), tumor necrosis factor-alfa (*TNF-*α**), and tumor necrosis factor-related apoptosis-inducing ligand *(TRAIL)*. Furthermore, in order to clarify whether the expansion of Tregs is a characteristic finding of chronic viral hepatitis, we examined *Foxp3* expression in liver biopsies of patients with other hepatic diseases, including nonalcoholic fatty liver disease (NAFLD), autoimmune hepatitis, primary biliary cirrhosis, and liver-toxicity induced by methotrexate. Correlations with the grade of inflammation and fibrosis were also investigated in order to identify its possible interaction with the above factors towards the progression of liver damage.

## 2. Material and Methods

### 2.1. Patients

Liver biopsy specimens obtained from 26 patients with chronic HBV hepatitis (CHB) (4 with cirrhosis) and 14 with chronic HCV hepatitis (CHC) were examined. Amongst CHB patients, 19 were newly diagnosed and 7 were responders under treatment for 48 weeks with Peg-IFNa2a or antivirals and relapse after treatment withdrawal for 24 weeks. No patient presented with coinfection with other hepatitis viruses (types A, D, and E) or superinfection with HIV. Results were compared with those obtained from the examination of biopsies from 11 patients with nonalcoholic fatty liver disease (NAFLD). Liver biopsies from 8 patients with autoimmune hepatic diseases (4 with autoimmune hepatitis and cirrhosis and 4 with primary biliary cirrhosis, PBC) and 2 patients with rheumatoid arthritis and methotrexate- (MTX-) related hepatotoxicity were also analyzed. None of the patients were receiving antiviral or immunomodulatory treatment during the last 3 months prior to liver sampling, except for the 2 patients with rheumatoid arthritis who were receiving MTX. Eight individuals submitted to liver biopsy due to a mild increase of aminotransferases but without liver necroinflammatory and architecture changes (histology negative for disease) served as controls. HBV DNA and HCV RNA quantification was performed by the use of the bDNA assay V2.0 (Bayer, Siemens) and the Cobas Amplicor system (Roche Molecular Systems), respectively. Demographic, clinicopathologic and serologic data of the 69 analyzed subjects are summarized in Table 1.

Each liver biopsy specimen was separated into two parts. One of them was immediately fixed in 10% formalin solution for diagnostic histological examination, and the other was snap frozen and stored at −80°C until further use. Formalin-embedded sections were stained by haematoxylin-eosin and Masson's trichrome. Two independent pathologists assessed and scored each biopsy, and discrepancies were further evaluated by an expert pathologist. In patients with viral hepatitis, the histological activity index (HAI) and the staging of fibrosis (0–6) in formalin-fixed tissues were assessed according to the modified HAI scoring by Ishak et al. [16]. In patients with NAFLD, the necroinflammatory grade and the fibrosis score (0–4) were assessed according to the scoring by Brunt et al. [17]. According to the intensity of liver inflammation biopsies, patients were classified as I-0 (absent inflammation), I-1 (minimal, HAI score 1–4 and mild grade inflammation for NAFLD), I-2 (mild, HAI score 5–8 and moderate-I grade with minimal portal inflammation for NAFLD), I-3 (moderate, HAI score 9–12 and moderate-II grade with marked portal inflammation for NAFLD), and I-4 (marked, HAI score 13–18 and severe grade for NAFLD) (Table 1). For patients with autoimmune hepatitis, the inflammation grade and fibrosis stage were estimated by morphological criteria similar with CHB and CHC patients and stratified accordingly, while, for patients with MTX-related hepatotoxicity, the inflammation grade and fibrosis stage were assessed and stratified similar to the patients with NAFLD (Table 1). However, patients with PBC, two of them with stage 1 and two with stage 3, according to Ludwig et al. [18] and Scheuer [19], were not estimated in the above stratification grades, because of the distinct pathological findings of the disease.

Informed consent was obtained by all participants, and the study was approved by the Institutional Review Board.

### 2.2. Quantitative Real-Time Reverse-Transcriptase PCR (qRT-PCR)

Total RNA was isolated from stored liver samples after homogenization, using TRI (Ambion, Austin, USA), according to manufacturer's instructions. cDNA was reversed transcribed from 1 *μ*g of the RNA, using a random 6-mer oligonucleotide primer (50 pmol/*μ*L) (Roche, USA) and M-MLV reverse transcriptase (Invitrogen, UK), according to manufacturer's instructions. 

The mRNA levels of ten genes, namely, *Foxp3, Fas, FasL, TRAIL, caspase-3, IL-10, TGF-β1, TNF-α*, interferon-gamma (*IFN-*γ**), and interleukin-1beta (*IL-1β*), were determined in a qRT-PCR reaction using Platinum-SYBR-Green PCR Supermix (Invitrogen, UK), in the automated thermocycler RotorGene 6000 (Corbett Life Science, Sydney, Australia). The beta-2-microglobulin (*B2M*) gene was used as an endogenous control for sample normalization (reference gene). An 1/20 aliquot of the cDNA reaction product was used in duplicate qRT-PCR reactions, and all measurements were averaged. Primers were commercially obtained by SABiosciences (Frederick, MD, USA). Thermocycler conditions for the *Foxp3, TGF-β1, caspase-3, TNF-α*, and *B2M* genes included an initial holding at 50°C for 2 min and subsequently at 95°C for 2 min, followed by 40 cycles at 95°C for 15 sec and 60°C for 60 sec. For the *Fas, FasL, IL-10*, and *IFN-*γ** genes, three-step PCR was performed with annealing at 55°C (for *Fas, FasL*, and *IL-10*) or 58°C (for *IFN-*γ**) for 15 sec and extension at 72°C for 30 sec (denaturation and cycles were similar as for the other genes). The efficiency of each qRT-PCR reaction ranged between 0.9 and 1.05. In order to verify the specificity of the PCR products, melting curve analysis was performed from 65°C to 95°C with 0.1°C/sec intervals and stepwise fluorescence acquisition. Relative quantification and calculation of the range of confidence were performed using the comparative ΔΔ^CT^ method, as described in [20]. The relative expression of each gene is presented as a multiple of the respective gene expression in the sample of a normal control, who presented with the lowest levels of aminotransferases. Expression data of this patient (expression = 1, for all genes) were, therefore, excluded from the statistical analysis.

### 2.3. Western Blot Analysis

The FOXP3 protein expression was determined in randomly selected samples from 7 patients with hepatic diseases (3 with CHB, 2 with CHC, and 2 with NAFLD) and 2 normal controls, using an anti-FOXP3 mouse monoclonal antibody (ab22510) from Abcam (Cambridge, UK). GAPDH (rabbit polyclonal, code: 2275-PC-100; Trevigen, Gaithersburg, Md, USA) served as a loading control, using an ultraviolet detection system (WesternDot 625 Goat Anti-Rabbit Western Blot Kit; Invitrogen), according to manufacturer's recommendations.

### 2.4. Statistical Analysis

For basic statistical calculations, *Foxp3, IL-10, TGF-β1, Fas, FasL, TRAIL, caspase-3, TNF-α* *, IFN-*γ*, and IL-1* *β* expression levels were treated as continuous variables. Differences of gene expression between disease groups were analyzed by the nonparametric Mann-Whitney *U* test. The association of the above parameters with inflammation and fibrosis grade was tested with Kruskal-Wallis H test. Sprearman's rank correlation coefficient was used to estimate the correlations of the expression among the aforementioned genes, as well as the correlations of gene expressions with aminotransferases levels or viral load. All statistical calculations were performed by the use of SPSS (version 16.0, Chicago, Il, USA). Differences were considered statistically significant when the *P* value (two sided) was <.05.

## 3. Results

### 3.1. Gene Expression in Relation to Liver Diseases

**Figure 1:**
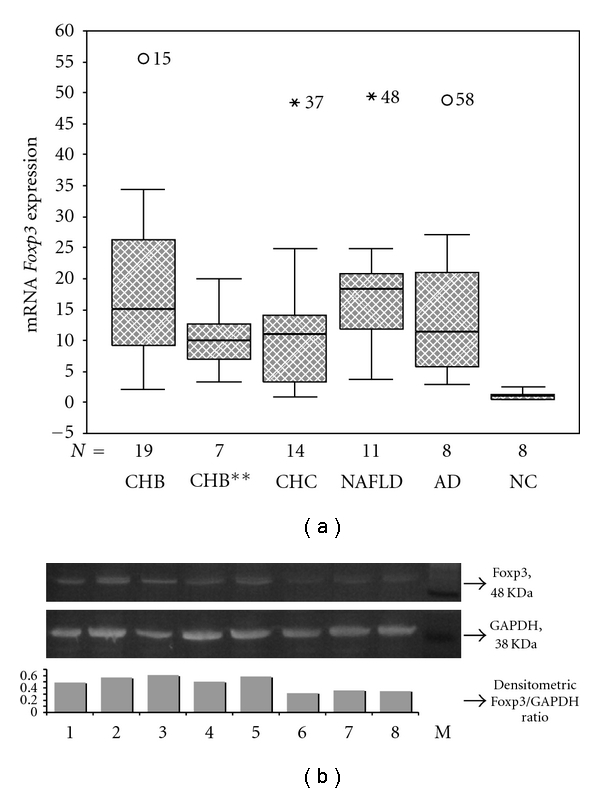
Expression of *Foxp3* in different hepatic diseases. (a) Boxplot diagram of the relative expression of *Foxp3* in the different disease subgroups (CHB: chronic HBV hepatitis, CHB**: chronic HBV hepatitis at relapse, CHC: chronic HCV hepatitis, NAFLD: non-alcoholic fatty liver disease, AD: autoimmune diseases/autoimmune hepatitis and primary biliary cirrhosis, and NC: normal controls). (b) Western blot analysis of the expression of FOXP3 and GAPDH. Lane 1: patient with CHC; Lanes 2 and 3: patients with CHB; Lane 4: patient with NAFLD; Lane 5: patient with autoimmune hepatitis; Lanes 6–8: normal controls; M: SeeBlue Plus2 Prestained Standard (Invitrogen, UK).

As shown in Table 2 and Figure 1, patients with CHB and CHC as well as those with the other hepatic diseases (NAFLD, autoimmune hepatitis, PBC, and MTX-related hepatotoxicity) presented a statistically significant increase of *Foxp3* mRNA levels compared to normal controls. In none of the patient groups, *Foxp3* expression correlated with the alanine aminotransferase (ALT) or aspartate aminotransferase (AST) levels and the viral load (serum HBV DNA or HCV RNA). The expression of FOXP3 was also confirmed by immunoblotting (Figure 1).

**Figure 2:**
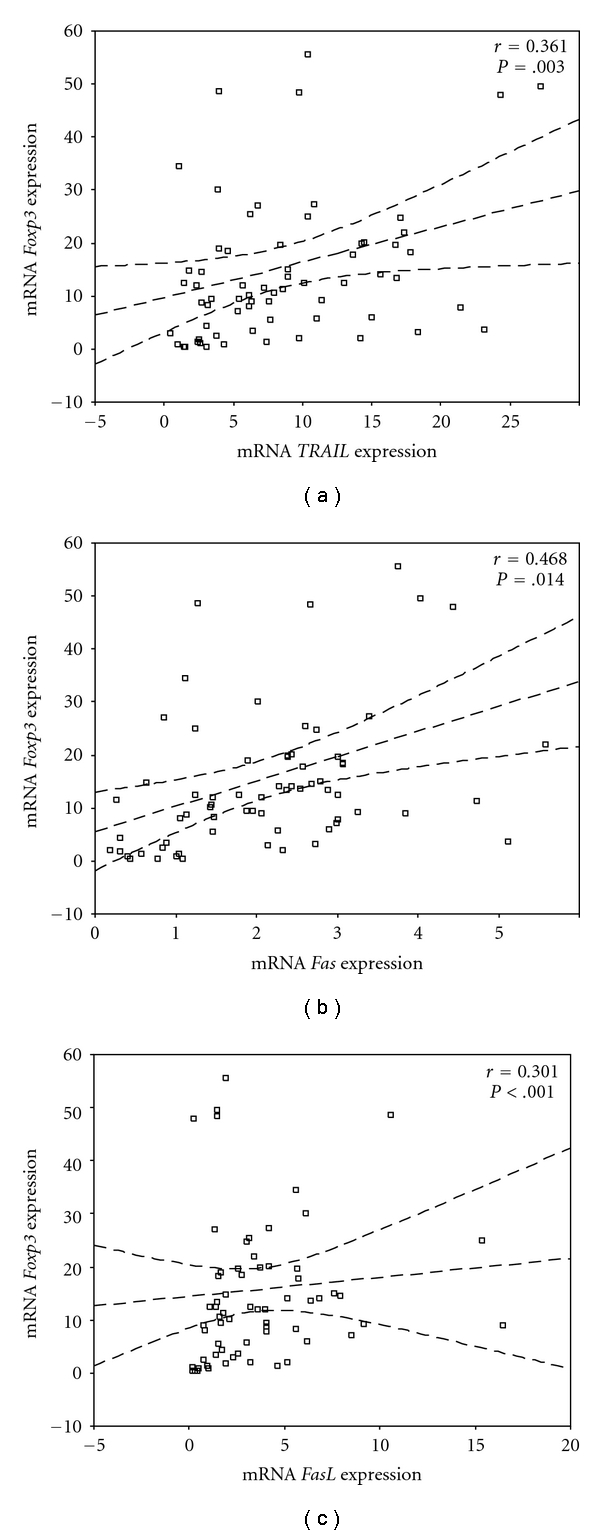
Results of the Spearman's rank correlation coefficient between *Foxp3* and *TRAIL* (a), *Foxp3* and *Fas* (b), and *Foxp3* and *FasL* (c), in all disease subgroups considered.

To ascertain whether the expression of specific apoptosis mediators was altered, the mRNA levels of *Fas, FasL, TRAIL, TNF-α*, and *caspase-3* were examined. A statistically significant increase of the mRNA expression of *FasL* was found in liver biopsies from all patient groups compared to normal controls (Table 2). Furthermore, *Fas* expression was found significantly increased in patients with CHB and NAFLD, while *TRAIL* expression was significantly increased in all patients, except of those with autoimmune diseases (Table 2). Positive correlations were observed between the intrahepatic expression levels of *Foxp3* with the expression of *Fas* (*P* =  .014), *FasL* (*P* <  .001) and *TRAIL* (*P* =  .003) (Figure 2), *Fas* and *TRAIL* (*P* <  .001), and *FasL* and *IFN-*γ** (*P* <  .001), irrespective of the cause of liver damage (viral, NAFLD, autoimmunity, MTX).

Patients with CHB and CHC displayed an approximately 3-fold significant decrease of *IL-10* expression compared to normal controls (Table 2). *IL-10* expression levels in these groups of patients were not significantly different from those of NAFLD patients. Compared to normal controls, NAFLD patients, as well as those with autoimmune hepatic diseases and MTX-related toxicity, presented with lower *IL-10* expression levels that, however, did not reach the level of statistical significance (Table 2). Moreover, *IL-1β* transcripts were significantly decreased than those of normal controls in the groups of CHB and CHC patients at diagnosis (Table 2).

No significant difference in the expression of *TGF-β1* was observed between the various groups of patients with liver diseases and normal controls (Table 2). However, a significant positive correlation between the expression levels of *TGF-β1* and those of *Fas, IL-10, TNF-α*, and *IL-1β* (*P* <  .05, in all cases) was found. 

To this point, it must be underlined that one CHB patient with very high *Foxp3* mRNA levels (25-fold higher than that of the reference sample) also displayed a high expression of *IL-10* and *TGF-β1* (approximately 6-fold and 4-fold higher than that of the reference sample, resp.). These findings were confirmed in repeated Q-RT-PCR analyses, and this patient did not differ from the other CHB patients, in any clinical or laboratory parameter. 

Finally, the expression levels of all genes in the posttreatment biopsies obtained from 7 CHB patients during the relapse of the disease did not differ from their expression in CHB patients at diagnosis.

### 3.2. Gene Expression in Relation to the Intensity of Inflammation and Fibrosis

Taking into account that no striking differences were observed between the various groups of patients with respect to the expression of the analyzed genes, we considered all subjects of the study as a whole group, in an attempt to investigate possible relations between the expression of the genes and the intensity of inflammation.

**Figure 3:**
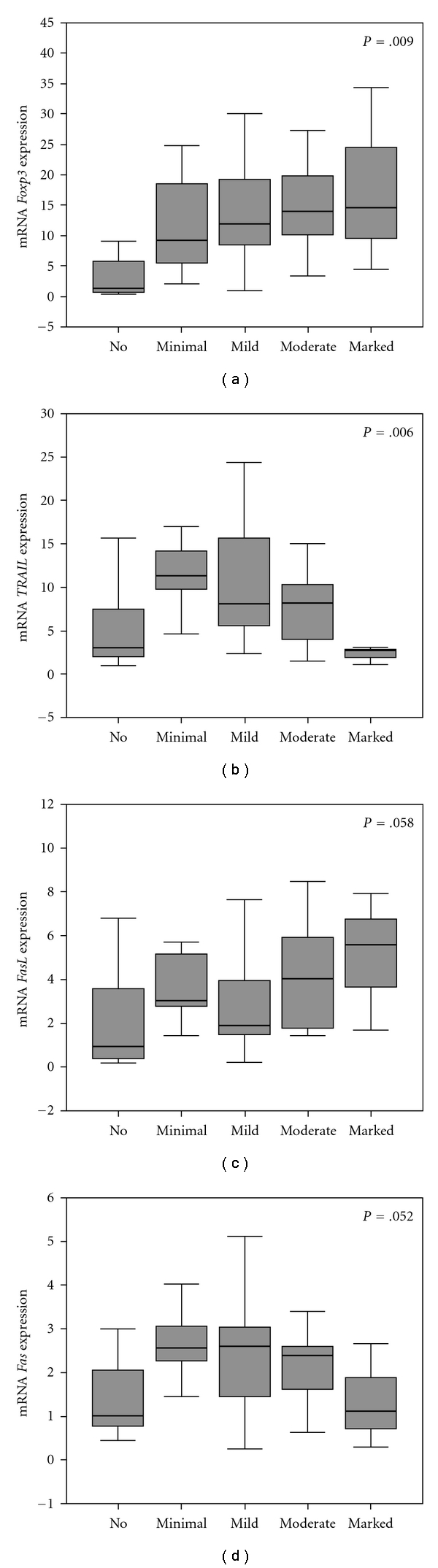
Boxplot diagrams presenting the expression of *Foxp3* and apoptosis mediators according to the intensity of liver inflammation (classification as presented in Section 2). Abscissa corresponds to relative mRNA expression. The pattern of expression in the *Foxp3* diagram followed also by *FasL*, but not by *Fas* and *TRAIL*. *P* value in each diagram refers to Kruskal-Wallis *H* test.

In relation to the intensity of inflammation, *Foxp3* exhibited a statistically significant increase of expression from normal liver to severe inflammation (Figure 3). This pattern of expression was nearly similar for *FasL* (Figure 3). On the other hand, *Fas* and *TRAIL* displayed a different pattern of expression, where, compared to I-0 subjects (without macroscopic evidence of inflammation), patients with minimal to moderate inflammation (I-1 to I-3) exhibited a statistically significant increase of expression that returned to those of I-0 subjects, as inflammation became severe (I-4) (Figure 3). *IL-10* and *TGF-β1* expression did not display any statistically significant change in relation to the intensity of inflammation. To this end, it must be noted that no patients with NAFLD presented with moderate or severe inflammation, that is, that all patients categorized as I-3 and I-4 represent those with chronic viral hepatitis and autoimmune cirrhosis (Table 1).

Moreover, a significant positive correlation between mRNA levels of *Foxp3*, *FasL*, and *IFN-*γ** with fibrosis (*P* =  .003, *P* =  .004, and *P* =  .013, resp.) was observed. Interestingly, fibrosis staging correlated negatively with the expression levels of *IL-10* (*P* =  .003) and *caspase-3* (*P* =  .013), but not with the expression of *TGF-β1* and other apoptosis mediators (*P* >  .05). Furthermore, a significant positive correlation between the expression levels of *IL-1β* and those of *TNF-α* and *caspase-3* was also observed (*P* <  .001 and *P* <  .001, resp.). 

Finally, as expected, HAI score was positively correlated with ALT levels (*P* =  .038), in patients with CHB and CHC.

## 4. Discussion

Our study provides clear evidence that irrespective of the cause of liver damage, *Foxp3* expression, and not *IL-10* and *TGF-β1*, appeared with a dramatic increase that relates to the intensity of liver inflammation. Moreover, apoptosis-induced inflammation is observed in a wide range of liver diseases.

It is well known that viruses sensitize hepatocytes to apoptosis whilst they are also capable of inhibiting it, in order to allow the survival of the infected host cells [21], as well as that increased expression of Fas/FasL appears in chronic viral hepatitis [22–27]. However, contradictory results exist in the literature regarding their relation with inflammation (HAI score) and ALT levels [26–28]. According to our results, a significant increase of *Fas* and *TRAIL* appears at the early stages of liver inflammation that could contribute to its induction. As the inflammation exacerbates and striking fibrosis (cirrhosis) is established, the expression of these apoptosis mediators declines. This finding can be attributed to the evolution of liver damage followed by destruction of hepatocytes and accumulation of lymphocytic infiltrate. As such, the elevated *FasL* expression, mainly expressed by CTLs, can also be explained, despite the fact that it is not followed by a parallel increase of *TRAIL* expression, as expected.

Most interestingly, however, our findings indicate that not only apoptosis is taking place in NAFLD, but also it can equally be responsible for the induction of inflammation, considered till now to be associated with lipid-related NF-*κ*B activation [28, 29]. Notwithstanding the limited cases examined with autoimmune hepatitis, PBC and MTX-hepatotoxicity, the same phenomenon was observed. Till now, there is only scarce evidence [30–33] that an increased expression of Fas/FasL accompanies NAFLD. The covariation of apoptosis mediators with inflammation that was observed in our NAFLD patients represents a line of supporting evidence to the results by Feldstein et al. [32], correlating inflammation with TUNEL-positive cells in this disease. 

As mentioned above, a high intrahepatic expression of another death ligand activating the caspase cascade and apoptosis, namely, *TRAIL*, was demonstrated. Although *TRAIL* induces apoptosis mostly in transformed cells, recent in vitro studies showed that it triggers steatosis and massive apoptosis in fresh liver explants from patients with viral hepatitis or fatty liver [34–37]. The increased intrahepatic expression of *TRAIL* that we found followed the same pattern of expression of *Fas*, indicating the significant role that this molecule might play in liver inflammation, irrespective of the cause. The nature (soluble or transmembrane) and the source (immune cells or hepatocytes) of *TRAIL* as well as the downstream molecules (receptors and mediators) activated during its signalling in hepatocytes or stellate cells remain to be determined. 

We demonstrated that the intensity of chronic liver inflammation was not associated with *IL-1β* expression, and its mRNA levels were significantly lower in patients with CHB and CHC compared to normal controls. Moreover, a strong positive correlation of the expression of proinflammatory cytokines IL-1*β* and TNF-*α* was also observed. Interestingly, our findings are in accordance with those of Bortolami et al., whereas a lower expression of *IL-1β* in patients with CHB and CHC compared to normal controls was also reported [38]. A plausible explanation could be the accumulation of lymphocyte infiltrate in chronic viral hepatitis, resulting in altered cellularity with a low proportion of cells producing the aforementioned cytokines. On the other hand, a low production of the above proinflammatory cytokines in chronic viral inflammation cannot be excluded. IL-1*β* and TNF-*α* are prototypic cytokines that exert pleiotropic effects on a variety of cells, playing a fundamental role in acute and chronic inflammatory conditions [39, 40]. Their role at the initial phases of local and systemic inflammation, triggering a complex network of signalling molecules, is indisputable and well characterized [39, 40]. Moreover, a sustained increased expression of IL-1*β* and TNF-*α* has been reported in several cases of chronic local inflammation, as in rheumatoid arthritis and inflammatory bowel diseases, where the anticytokine therapy is very effective, reducing symptoms and slowing or arresting tissue damage [41]. However, in cases of severe systemic inflammation, a downregulation of mRNA levels of the proinflammatory cytokines *IL-1β* and *TNF-α*, produced by liver and blood cells, has been reported [41, 42]. As a result, the anticytokine therapy for such patients is detrimental [41]. Our findings may display that a similar phenomenon is observed in chronic liver inflammation, especially in cases of chronic viral hepatitis. Thus, considering the sustained low levels of the proinflammatory cytokines in patients with chronic viral hepatitis as well as the fact that the anticytokine therapy for rheumatoid arthritis and inflammatory bowel diseases can also result in reactivation of HBV or HCV infections [43], the contribution of an inappropriate inflammatory reaction as the causative of the chronicity of viral infections should be taken into account and remains to be determined. 

Our results, regarding *Foxp3, IL-10*, and *TGF-β1* expression, imply that nTregs, and not iTregs, could contribute by processes unknown as yet to inflammatory liver disease. Most intriguing is the finding that increased *Foxp3* expression, and therefore nTregs intrahepatic accumulation, characterizes not only viral hepatitis but also to an equal degree, NAFLD as well as autoimmune hepatitis, PBC and MTX-related hepatotoxicity. Till now, the increased frequency of nTregs in the peripheral blood and/or their accumulation in the liver of patients with CHB or CHC has been attributed to the expansion of Tregs modulating the function of virus-specific T cells [8–11, 13]. Moreover, it has been postulated that viruses contribute to the production of virus-specific Tregs, suppressing the virus-specific T cell clones and thus allowing the persistence of viral infections [6–8, 11]. However, evidence supporting this hypothesis remains obscure. On the contrary, it has been described that in mice, the depletion of nTregs leads to immunopathology and deterioration of infectious diseases induced by various pathogens [44, 45]. Indeed, Wei et al. demonstrated that the depletion of FOXP3^+^ Tregs results in fulminant hepatitis in a mouse model of immune-mediated liver damage (induced by Concanavalin A) [46]. According to our results, persistent liver inflammation, regardless of its cause, seems to represent a main factor that contributes to the expansion of nTregs. Should this be the case, then the described suppression of virus-specific T cells could be considered as a bystander effect of the nTregs that have been expanded due to the persistent apoptosis-induced inflammation. 

Taken collectively, our results are in line with the attractive view of Zheng and Rudensky claiming that Tregs “have a vital role in preventing autoimmunity and pathology inflicted by uncontrolled immune responses to infections” [1]. As such, the evidence provided by our work can be integrated in a comprehensive protective model as detailed below. The excessive apoptosis induced by various tissue insults may overcome the capability of macrophages to safely clear the apoptotic cells. Consequently, the emergence of inflammation cannot be prevented by the secretion of anti-inflammatory cytokines from macrophages. In parallel, excessive autoantigen presentation is taking place leading to the activation of autoreactive T cells. Pre existing nTreg clones are therefore expanded, in order to prevent the self-tissue damage and to avoid catastrophic pathology.

Recent animal and human studies demonstrated that intrahepatic Tregs were increased in autoimmune liver diseases [47, 48], considering also that in AIH they were found fewer than in PBC, estimated by immunocytochemistry [47]. Despite the fact that our work provides also limited, yet consistent, data with regard to the autoimmune hepatitis/cirrhosis and PBC, it can be postulated that, in these cases, apoptosis-induced inflammation might be the result, rather than the cause, of the autoimmune damage that might have initiated due to a defective nTreg function. This being the case, the above initiates and perpetuates a vicious cycle leading to the destruction of self-tissues. In other words, our model indicates that Tregs could be the missing link to the “waste disposal” hypothesis of autoimmunity [49].

Should a similar protective role of nTregs be uncovered in other types of apoptosis-induced inflammation, then the proposed targeting of apoptosis [50], rather than of nTregs, could prove to be a more promising therapeutic modality. Our results and the pathophysiological model we propose confirm that, possibly with the exception of autoimmunity, nTregs represent a protective mechanism whose manipulation should be carefully considered. However, further research towards the elucidation of the underlying casual relationships is required, in order to clarify whether our findings signify the existence of a uniform Treg-mediated regulatory mechanism of apoptosis-induced inflammation.

## Figures and Tables

**Table 1:** Clinicopathological and serological data of the patients of the study.

	Normals	Chronic HBV hepatitis		Chronic HCV hepatitis	NAFLD^a^	Autoimmune diseases^b^	MTX-related toxicity^c^
		Diagnosis	Relapse				
No.	8	19	7	14	11	8	2
Sex (M/F)^d^	5/3	9/10	4/3	11/3	6/5	2/6	0/2
Age (years) (mean, range)	53.3 (27–67)	49.3 (24–64)	48.6 (22–65)	39.6 (27–50)	41.9 (21–66)	56.8 (37–73)	66 (60–72)
AST (U/*μ*L)^e^ (mean, range)	46.6 (42–56)	185 (17–1969)	76.6 (31–277)	69.5 (24–218)	34.9 (19–63)	66.5 (30–225)	32.5 (24–41)
ALT (U/*μ*L)^f^ (mean, range)	55 (48–70)	193 (15–1478)	102 (32–332)	91.2 (32–213)	58.8 (15–141)	65.5 (31–212)	28.5 (17–40)

Inflammation grade^g^							
I-0	8	—	—	—	3	—	—
I-1	—	4	—	2	3	—	2
I-2	—	8	6	6	5	—	—
I-3	—	5	1	6	—	3	—
I-4	—	2	—	—	—	1	—

Fibrosis^g^ (mean, range)	0	3.4 (0–6)	3.4 (1–5)	2.3 (1–4)	0.5 (0–2)	2.8 (0–6)	1 (1–1)
HAI score (mean, range)		7.52 (1–15)	7.57 (5–11)	7.21 (2–12)			
Viral load (mean, range)		79.8 Meq/mL (<0.01–21)	6.39 Meq/mL (<0.01–4.5)	1.16 × 10^6^ IU/mL (0.06–6.2)			

^a^NAFLD: nonalcoholic fatty liver disease; ^b^autoimmune diseases refers to autoimmune hepatitis (4 patients) and primary biliary cirrhosis (4 patients); ^c^MTX: methotrexate; ^d^M: male; F: female; ^e^AST: aspartate aminotransferase; ^f^ALT: alanine aminotransferase; ^g^inflammation grade (I-0: without inflammation, I-1: minimal, I-2: mild, I-3: moderate, and I- 4: marked) and fibrosis stage were assessed as presented in Section 2.

**Table 2:** Relative expression of the examined genes.

Gene	Normals	Chronic HBV hepatitis		Chronic HCV hepatitis	NAFLD^b^	Autoimmune diseases^c^	MTX-related toxicity^d^
	(no. 8)	Diagnosis (no. 19)	Relapse (no. 7)	(no. 14)	(no. 11)	(no. 8)	(no. 2)
	Mean ± S.D.	Mean ± S.D. (*P* value^a^)	Mean ± S.D. (*P* value^a^)	Mean ± S.D. (*P* value^a^)	Mean ± S.D. (*P* value^a^)	Mean ± S.D. (*P* value^a^)	Mean ± S.D.

*Foxp3*	1.11 ± 0.71	20.3 ± 16.0 (**<.001**)	10.4 ± 5.47 (**.001**)	12.7 ± 12.3 (**.001**)	18.4 ± 11.9 **<.001**	16.0 ± 15.2 (**.001**)	28.4 ± 27.8
*TGF-β* **1**	1.56 ± 1.49	1.10 ± 0.84 (.710)	0.53 ± 0.31 (.053)	0.73 ± 0.58 (.065)	1.60 ± 1.01 (.620)	1.43 ± 1.04 (.834)	0.72 ± 0.17
*IL-10*	1.50 ± 1.02	0.66 ± 1.33 **(.011)**	0.20 ± 0.24 **(.016)**	0.48 ± 0.77 **(.017)**	0.79 ± 0.77 (.131)	0.64 ± 0.94 (.093)	0.59 ± 0.62
*Fas*	0.82 ± 0.24	2.25 ± 0.70 **(<.001)**	2.12 ± 0.78 **(.003)**	1.55 ± 1.07 (.179)	3.51 ± 1.17 **(<.001)**	1.56 ± 0.94 (.132)	4.14 ± 0.41
*FasL*	1.05 ± 1.47	4.20 ± 2.05 **(.001)**	3.05 ± 1.98 **(.028)**	4.20 ± 4.09 **(.004)**	3.94 ± 4.42 **(.004)**	4.33 ± 3.69 **(.008)**	0.49 ± 0.39
*TRAIL*	2.89 ± 2.04	7.28 ± 4.34 **(.007)**	11.3 ± 6.29 **(.015)**	8.86 ± 4.56 **(.001)**	15.3 ± 6.64 **(<.001)**	3.52 ± 2.11 (.355)	15.3 ± 12.7
*Caspase-3*	1.87 ± 1.85	1.13 ± 0.74 (.915)	0.76 ± 0.43 (.366)	1.66 ± 1.34 (.700)	1.97 ± 1.05 (.283)	3.19 ± 2.23 (.093)	2.28 ± 0.66
*TNF-α*	3.43 ± 4.70	1.77 ± 3.12 (.307)	1.54 ± 1.93 (.201)	2.73 ± 3.58 (.544)	8.17 ± 11.0 (.480)	6.63 ± 9.11 (.186)	1.21 ± 0.93
*IFN-*γ**	1.92 ± 1.77	5.47 ± 5.99 (.202)	4.17 ± 2.46 (.186)	2.19 ± 1.77 (.628)	4.18 ± 4.93 (.572)	8.43 ± 7.25 (.059)	0.37 ± 0.26
*IL-1β*	1.37 ± 1.05	0.51 ± 1.39 **(.022)**	0.41 ± 0.66 (.055)	0.33 ± 0.26 **(.042)**	0.99 ± 0.95 (.322)	1.15 ± 1.12 (.571)	0.30 ± 0.23

^a^Statistical significance refers to comparison with the expression levels in the normal controls (Mann-Whitney *U* test); ^b^NAFLD: nonalcoholic fatty liver disease; ^c^autoimmune diseases group consists of 4 patients with autoimmune hepatitis and 4 with primary biliary cirrhosis; ^d^MTX: methotrexate.
